# Identification of VWA5A as a novel biomarker for inhibiting metastasis in breast cancer by machine-learning based protein prioritization

**DOI:** 10.1038/s41598-024-53015-1

**Published:** 2024-01-30

**Authors:** Jiwon Koh, Dabin Jeong, Soo Young Park, Dohyun Han, Da Sol Kim, Ha Yeon Kim, Hyeyoon Kim, Sohyeon Yang, Sun Kim, Han Suk Ryu

**Affiliations:** 1 Department of Pathology, Seoul National University Hospital, Seoul National University College of Medicine, 101 Daehakro, Seoul, 03080 South Korea; 2 Cancer Research Institute, Seoul National University, Seoul, South Korea; 3 Interdisciplinary Program in Bioinformatics, Seoul National University, Seoul, South Korea; 4 Department of Pathology, Seoul National University College of Medicine, Seoul, South Korea; 5 Proteomics Core Facility, Biomedical Research Institute, Seoul National University Hospital, Seoul, South Korea; 6 Transdisciplinary Department of Medicine & Advanced Technology, Seoul National University Hospital, Seoul, South Korea; 7 Department of Computer Science and Engineering, Institute of Engineering Research, Seoul National University, Gwanak-ro 1, Seoul, 08826 South Korea; 8 Pharmonoid Co., Ltd., Seoul, South Korea

**Keywords:** Cancer, Breast cancer

## Abstract

Distant metastasis is the leading cause of death in breast cancer (BC). The timing of distant metastasis differs according to subtypes of BCs and there is a need for identification of biomarkers for the prediction of early and late metastasis. To identify biomarker candidates whose abundance level can discriminate metastasis types, we performed a high-throughput proteomics assay using tissue samples from BCs with no metastasis, late metastasis, and early metastasis, processed data with machine learning-based feature selection, and found that low VWA5A could be responsible for shorter duration of metastasis-free interval. Low expression of *VWA5A* gene in METABRIC cohort was associated with poor survival in BCs, especially in hormone receptor (HR)-positive BCs. In-vitro experiments confirmed tumor suppressive effect of VWA5A on BCs in HR+ and triple-negative BC cell lines. We found that expression of VWA5A can be assessed by immunohistochemistry (IHC) on archival tissue samples. Decreasing nuclear expression of VWA5A was significantly associated with advanced T stage and lymphatic invasion in consecutive BCs of all subtypes. We discovered lower expression of VWA5A as the potential biomarker for metastasis-prone BCs, and our results support the clinical utility of VWA5A IHC, as an adjunctive tools for prognostication of BCs.

## Introduction

Breast cancer (BC) is a heterogeneous disease with respect to its clinicopathological features and molecular biologic profiles^1^. Though novel therapeutics and prognostic classifiers have emerged during the last decade, BC remains the leading cause of cancer-related deaths worldwide, and it is the most lethal type of cancer among South Korean women^2^. It is widely known that even optimally treated BC patients carry the risk of relapse after 5 years from diagnosis and that estrogen receptor-positive (ER+) and luminal A patients are at the highest risk for late-onset metastasis^3^.

Various prognostic tools are currently available for the selection of patients eligible for adjuvant therapy and prediction of prognosis, including Recurrence Score derived by Oncotype DX^4^ or Mammaprint^5^. However, the relatively high cost of these gene expression assays hinders the public accessibility of tools, especially for patients with BC residing outside of the United States or Europe. Moreover, though these tools are highly valuable in predicting the patients with recurrence of distant metastasis within the first 5 years after diagnosis, their usefulness for prediction of late metastasis is limited. These suggest that there is a critical need for the identification of novel, robust prognostic biomarkers with excellent performance for prediction of both early and late metastasis^6,7^.

The discovery of predictive markers for early and late metastasis can provide significant guidance toward the proper management of patients with BC^8^. For example, identifying the patients carrying higher risk for metastasis would result in more active surveillance, while those with lower risk could be spared from unnecessary adjuvant treatments and resultant side effects.

High-throughput proteomics assay enables the massive screening for ideal biomarkers in cancer research, by quantitative and qualitative analyses of a wide range of candidate peptides^9^. The initial discovery from the proteomics assay can be translated into clinical practice after functional validation. For example, a recent study used liquid chromatography with tandem mass spectrometry (LC–MS/MS) on formalin-fixed paraffin-embedded (FFPE) BC samples to discover protein biomarkers for prediction of chemotherapy response. The candidate markers were subject to in vitro functional analysis using BC cell lines after knockdown experiments^10^. From the pathologists’ point of view, proteomics-driven search of candidate biomarkers followed by immunohistochemistry (IHC)-based validation can lead to the development of robust, clinically applicable assay.

We hypothesized that the baseline protein expression present within the tumor at the time of initial diagnosis may predict the development of late or early metastasis during the clinical course. Therefore, we established a discovery set of patients who were initially diagnosed with non-metastatic BC; a subset of the patients developed early or late metastasis. Then we designed the high-throughput proteomics assay to explore the protein expression profiles using initially resected BC samples and sought to compare the protein expression profiles of BC with no metastasis (NM), late metastasis (LM), and early metastasis (M). We then performed machine learning-based feature selection to identify biomarker candidates whose abundance level can discriminate the metastasis types and found that loss of VWA5A could be responsible for the shorter duration of metastasis-free interval. We then validated the role of VWA5A in BC metastasis through in vitro functional study, followed by IHC on a large cohort of BC.

## Results

### Clinicopathological characteristics of discovery set

A total of 29 patients were included in the discovery set (Table 1). Ten patients had no metastasis (NM group), 9 patients had LM, and 10 patients were in the M group. Mean metastasis-free survival (MFS) time was 8.4 years with LM, and 1.6 years in the patients in M group.

**Table 1:** Clinicopathological characteristics of the discovery set.

	NM	LM	M	Total	*p*
Age (mean ± SD)	46 ± 6	46 ± 14	47 ± 12	46 ± 11	0.776
MFS (mean ± SD)	NA	8.4 ± 3.1	1.6 ± 0.6	4.8 ± 4.1	< 0.001
Nuclear grade					0.115
1	0 (0.0%)	1 (11.1%)	0 (0.0%)	1 (3.4%)	
2	5 (50.0%)	4 (44.4%)	1 (10.0%)	10 (34.5%)	
3	5 (50.0%)	4 (44.4%)	9 (90.0%)	18 (62.1%)	
Histologic grade					0.070
II	5 (50.0%)	5 (55.6%)	1 (10.0%)	11 (37.9%)	
III	5 (50.0%)	4 (44.4%)	9 (90.0%)	18 (62.1%)	
ER IHC					0.377
Negative	4 (40.0%)	3 (33.3%)	6 (60.0%)	13 (44.8%)	
Positive	6 (60.0%)	6 (66.7%)	4 (40.0%)	16 (55.2%)	
PR IHC					0.185
Negative	4 (40.0%)	5 (55.6%)	7 (70.0%)	16 (55.2%)	
Positive	6 (60.0%)	4 (44.4%)	3 (30.0%)	13 (44.8%)	
HER2 status					1.000
Negative	8 (80.0%)	8 (88.9%)	8 (80.0%)	24 (82.8%)	
Positive	2 (20.0%)	1 (11.1%)	2 (20.0%)	5 (17.2%)	
Subtype					0.137
ER+/HER2−	6 (60.0%)	6 (66.7%)	3 (30.0%)	15 (51.7%)	
ER−/HER2+	2 (10.0%)	1 (11.1%)	2 (20.0%)	4 (13.8%)	
TNBC	2 (20.0%)	2 (22.2%)	5 (50.0%)	10 (34.5%)	
pT stage					1.000
pT1	1 (10.0%)	2 (22.2%)	1 (10.0%)	4 (13.8%)	
pT2	9 (90.0%)	7 (77.8%)	9 (90.0%)	25 (86.2%)	
pN stage					1.000
pN0	10 (100.0%)	8 (88.9%)	10 (100.0%)	28 (96.6%)	
pN1	0 (0.0%)	1 (11.1%)	0 (0.0%)	1 (3.4%)	
Stage					1.000
I	1 (10.0%)	2 (22.2%)	1 (10.0%)	4 (13.8%)	
II	9 (90.0%)	7 (77.8%)	9 (90.0%)	25 (86.2%)	
Total	10 (34.5%)	9 (31.0%)	10 (34.5%)	29 (100.0%)	

*NM* no metastatsis, *LM* late metastasis, *M* metastasis, *SD* standard deviation, *MFS* metastasis free survival, *IHC* immunohistochemistry, *TNBC* triple negative breast cancer.

Clinicopathological characteristics among the three groups—NM, LM, and M—were compared, where no significant differences in patients’ age, histologic grade, BC subtype, or pathologic stage were observed. The majority of the patients (86.2%; 25/29) were stage II, and only one patient in the LM group initially had lymph node metastasis (3.4%; 1/29).

### Proteomic profiles of the discovery set

A total of 9455 proteins were quantified across the discovery set, and 6639 proteins were quantified with label-free quantification (LFQ) intensities after filtration and imputation. Differentially expressed proteins (DEPs) were identified, where 830 proteins were found to be differentially expressed among the groups with an false discovery rate (FDR)-adjusted *p-*value < 0.1 (Supplementary Table S2). Among the DEPs, we found that the expression of VWA5A sequentially decreased from the NM group (median LFQ, 29.6; min–max, 28.9–30.3), LM group (median LFQ, 29.2; min–max, 28.0–28.3), and M group (median LFQ, 28.2; min–max, 27.2–29.3) (Supplementary Fig. S1).

**Figure 1:**
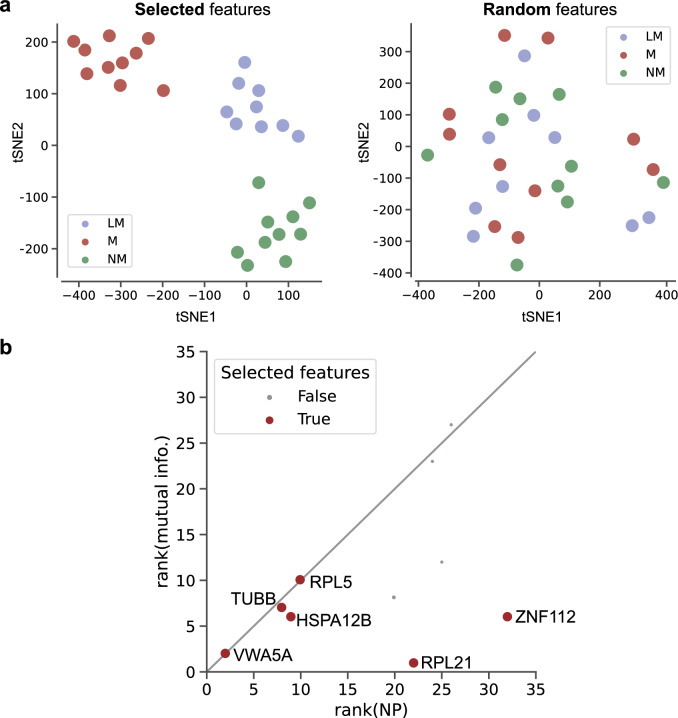
VWA5A is prioritized as a biomarker candidate for metastasis type discrimination. (**A**) The t-SNE plots of the patient embeddings with the nine features selected by our method (left) and with randomly selected features. (**B**) Scatter plots of the nine candidates in terms of mutual information ranks and NP score ranks. Candidate biomarkers selected by our method are marked as red dots.

To further characterize the implication of DEPs, we went through the recursive feature addition experiment, until the prediction performance of the support vector machine (SVM) classifier reached 0.8 precision, 0.79 recall, and 0.79 f1 score (Supplementary Fig. S2). After each of 29 leave-one-out cross-validation (LOOCV) experiments, a different set of features were selected. The nine biomarker candidates were finally chosen as those that were selected in at least 3 LOOCV experiments, which included VWA5A. The t-SNE embeddings of patients with the nine biomarker candidates could clearly discriminate metastasis type (Fig. 1A). The nine makers showed significantly high mutual information (MI) and network propagation (NP) scores compared to the other proteins (Supplementary Fig. S3), and when considering the combined ranks in MI and NP score, VWA5A outstood as the highest among the nine biomarker candidates, 2nd in the MI ranking and 1st in the NP ranking (Fig. 1B, Supplementary Fig. S3).

### External validation using publicly available gene expression data

**Figure 2:**
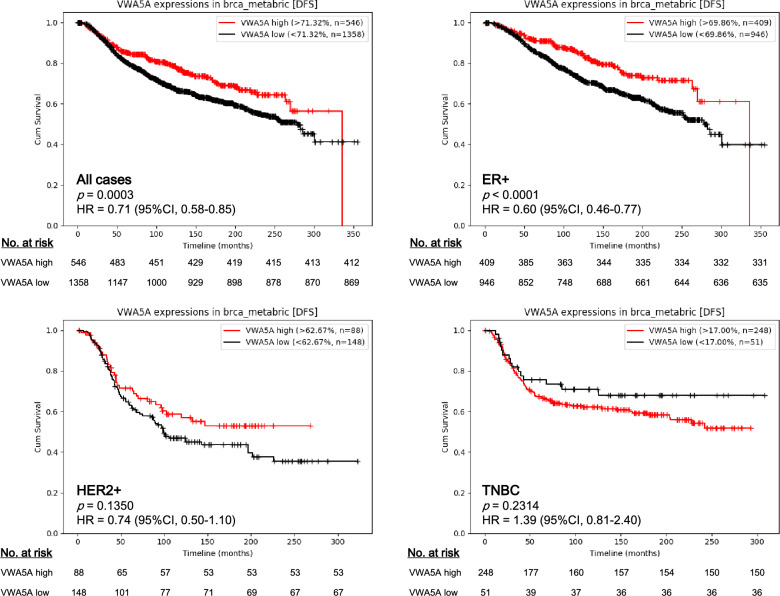
Prognostic relevance of *VWA5A* gene expression on disease free survivals of patients in METABRIC cohort. Higher *VWA5A* expression was significantly associated with favorable disease free survivals in METABRIC cohort.

Via Cancer Target Gene Screening (CTGS), we sought to assess the prognostic significance of *VWA5A* expression in the Molecular Taxonomy of Breast Cancer International Consortium (METABRIC) dataset. We compared the disease free survival (DFS) of the *VWA5A*-high group and *VWA5A*-low group, using the cut-off determined by Cutoff Finder application. We found that high *VWA5A* expression is a strong, significant favorable prognostic factor for DFS in all cases with BC in METABRIC cohort (n = 1904; *p* = 0.0001; hazard ratio [HR] 0.71; 95% confidence interval [CI] 0.58–0.85; Fig. 2). The favorable prognostic performance of high *VWA5A* expression was maintained within the ER+ subgroup (n = 1355; *p* < 0.0001; HR 0.60; 95% CI 0.46–0.77) but not in the HER2+ subgroup (n = 236; *p* = 0.1350; HR 0.75; 95% CI 0.50–1.10) and in TNBC subset (n = 299; *p* = 0.2314, HR 1.39; 95% CI 0.81–2.40). The Harrell’s concordance index (c-index) of VWA5A in predicting DFS was 0.543 in the total population, 0.545 in ER+ subgroup, 0.525 in HER2 + subgroup, and 0.518 in TNBC.

The prognostic significance of *VWA5A* on overall survival (OS) was also confirmed in all cases with BC (*p* = 0.0009; HR 0.82; 95% CI 0.73–0.92) and ER+ BCs (*p* = 0.0003; HR 0.74; 95% CI 0.62–0.87), but not in HER2+ and TNBC cases (Supplementary Fig. S4). The c-index of *VWA5A* expression for OS prediction in all BC cases and ER + BCs were 0.530 and 0.536, respectively.

To cross-check the results of the external validation, we assessed the relationship between LFQ values of VWA5A and the DFS of patients in the discovery set. We found the significant prognostic impact of VWA5A protein levels in discriminating DFS in the discovery set (*p* < 0.0001; HR 0.18; 95% CI 0.08–0.38).

### Biological role of VWA5A assessed by in vitro functional assay

Strong prognostic significance of VWA5A expression found in the pooled analysis METABRIC database, but not in certain subtypes of BCs indicate that this molecule may behave differently according to the intrinsic subtypes. Therefore, we sought to assess whether the knockdown of these genes can affect the biological behavior in an HR+ cell line (T47D) and two TNBC cell lines (BT20 and HCC70).

**Figure 3:**
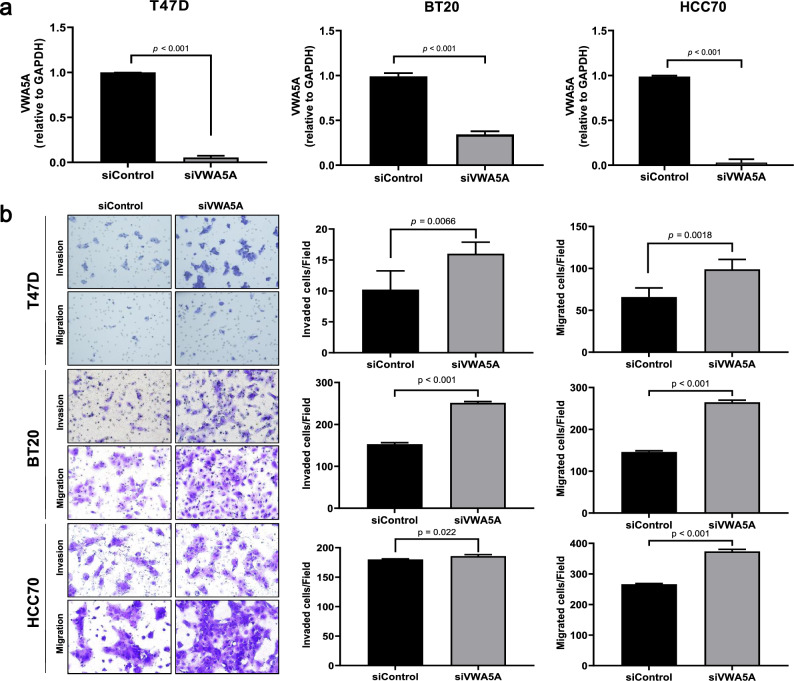
Biological role of VWA5A assessed by in vitro functional assay. (**A**) Knock-out *VWA5A* using siRNA transfection resulted in marked decrease in the expression levels of *VWA5A*. (**B**) siRNA transfected cell lines T47D, BT20 and HCC70 showed more aggressive behaviors including increased cellular invasion and migration.

After transfection with siVWA5A, T47D, BT20, and HCC70 cell lines showed marked reduction of VWA5A expression assessed by RT-PCR (Fig. 3A). We also confirmed showed stronger propensity toward invasion and migration after transfection with siRNA against VWA5A in all three of the experimented cell lines (Fig. 3B). No morphological changes were observed after knockout of VWA5A.

To analyze the effect of expression of the gene VWA5A on cell proliferation, breast cancer cell lines were divided into subtypes, and cell proliferation and protein expression levels were confirmed (Supplementary Fig. S5). In the luminal (MCF7 and T47D) and TNBC type cell lines (BT20, HCC1143, HCC1937, MDA-MB-231, MDA-MB-468 and HCC1395), the lower the VWA5A expression, the faster the cell proliferation. On the other hand, in the HER2 type, the higher the VWA5A protein expression, the faster the cell proliferation. These results demonstrate that lower protein expression of VWA5A leads to faster cell proliferation in luminal and TNBC cells, suggesting that VWA5A acts as a tumor suppressor and that the expression of VWA5A is inversely correlated with cell proliferation.

Interestingly, cell proliferation assay after transfection of the T47D line with siVWA5A showed that the cellular proliferation capability is reduced compared to the control (Supplementary Fig. S5c). This suggests that at least in HR+ BC cells, increased invasive and migratory behavior after VWA5A knockdown may be the result of increased cell-intrinsic metastatic potential, not explainable by proliferative capability alone. Taken together, these in vitro functional analyses confirmed the tumor suppressive effect of VWA5A on BCs especially regarding invasive and migratory potentials, therefore, supporting our discovery on proteomics-driven biomarker screening.

### Clinicopathological characteristic of BCs with high VWA5A expression

**Figure 4:**
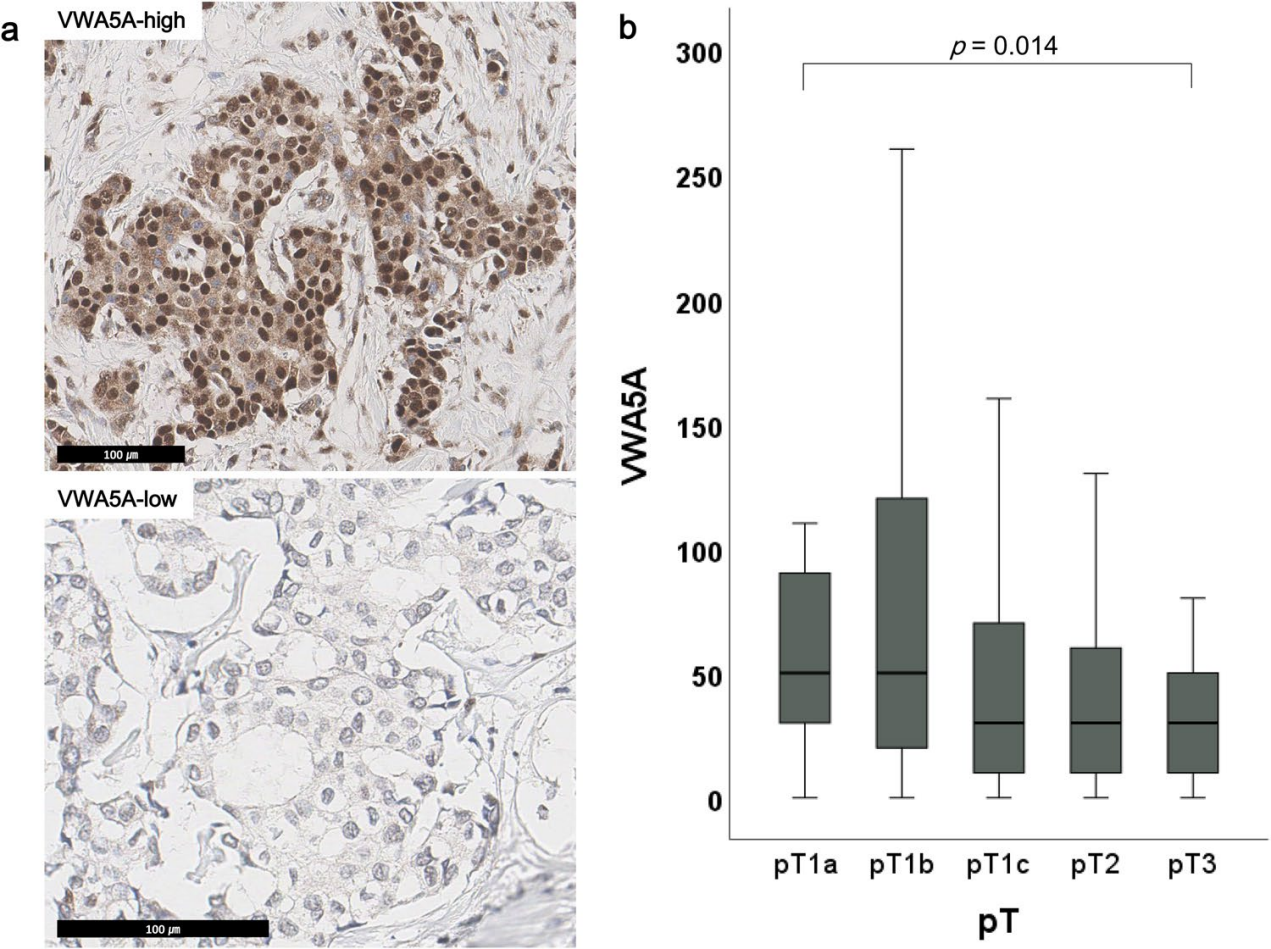
Expression of VWA5A in the validation set. (**A**) VWA5A expression was assessed by immunohistochemistry and histoscore of 50 was used to discriminate VWA5A-low and VWA5A-high groups. (**B**) Lower VWA5A expression of breast cancer cells were associated with advance pT stage of the patients in the validation set.

To validate the biological significance of VWA5A expression on BC, we performed IHC for VWA5A on the tissue microarrays (TMA) composed of surgically resected BC tissue samples from the validation set. Of the 1003 patients in the validation set, 966 (96.3%) samples had available tumor cells on TMA for adequate interpretation of VWA5A IHC results. 42.7% of the patients were classified as VWA5A-high group, while 57.3% were VWA5A-low (Fig. 4A).

Various clinicopathological features were compared between VWA5A-low and VWA5A-high groups and summarized in Table 2. We found that patients in the VWA5A-low group were more likely to be diagnosed with higher pT stages (*p* = 0.028; linear-by-linear test), and the decrease in the H-scores of VWA5A was associated with advanced T stage in the consecutive BC population (*p* = 0.014; Kruskal–Wallis test; Fig. 4B). In addition, BCs with lower expression of VWA5A were more likely to show lymphatic invasion (*p* = 0.009; Table 2). Though no significant association between VWA5A expression and pN stage was seen in the total consecutive BC validation set (*p* = 0.481), lower VWA5A expression was associated with positive pN stage within HR+/HER2− subgroup (*p* = 0.036; Supplementary Table S3). Regarding the patients’ survival, we found no significant differences in MFS (*p* = 0.125; HR 0.68; 95% CI 0.41–1.12) and OS (*p* = 0.089; HR 0.40; 95% CI 0.13–1.20). Though survival differences were not found, distinct clinicopathological features of the IHC-defined VWA5A-low group in the validation set support the aggressive behavior of VWA5A-low tumors and suggest the clinical implication of VWA5A expression on the BCs.

**Table 2:** Clinicopathological characteristics according to VWA5A expression in the validation set.

	VWA5A-low	VWA5A-high	Total	*p*
Age (mean ± SD)	51 ± 11	50 ± 10	51 ± 10	
Subtype				0.789
HR+HER2−	330 (59.6%)	246 (59.7%)	576 (59.6%)	
HR+HER2+	20 (7.2%)	30 (7.3%)	70 (7.2%)	
HR−HER2+	43 (7.8%)	40 (9.7%)	83 (8.6%)	
TNBC	98 (17.7%)	64 (15.5%)	162 (16.8%)	
Unknown	43 (7.8%)	32 (7.8%)	75 (7.8%)	
Nuclear grade				0.324
1	10 (1.8%)	7 (1.7%)	17 (1.8%)	
2	222 (40.1%)	152 (36.9%)	374 (38.7%)	
3	322 (58.1%)	253 (61.4%)	575 (59.5%)	
Histologic grade				0.704
I	31 (5.6%)	22 (5.4%)	53 (5.5%)	
II	211 (38.2%)	165 (40.1%)	376 (39.0%)	
III	311 (56.2%)	224 (54.5%)	535 (55.5%)	
Lymphatic invasion				0.009
Absent	335 (60.8%)	280 (69.0%)	615 (64.3%)	
Present	216 (39.2%)	126 (31.0%)	342 (35.7%)	
Distant metastasis				0.183
Absent	524 (94.6%)	381 (92.5%)	905 (93.7%)	
Present	30 (5.4%)	31 (7.5%)	14 (1.4%)	
pT stage				0.028
pT1a	3 (0.5%)	7 (1.7%)	10 (1.0%)	
pT1b	26 (4.7%)	36 (8.7%)	62 (6.2%)	
pT1c	234 (42.2%)	170 (41.3%)	404 (41.8%)	
pT2	273 (50.2%)	187 (45.4%)	465 (48.1%)	
pT3	13 (2.3%)	12 (2.9%)	25 (2.6%)	
pN stage				0.481
pN0	355 (64.2%)	275 (67.4%)	630 (65.6%)	
pN1	143 (25.9%)	95 (23.3%)	238 (24.8%)	
pN2	38 (6.9%)	24 (5.9%)	62 (6.5%)	
pN3	17 (3.1%)	14 (3.4%)	31 (3.2%)	
Total	554 (57.3%)	412 (42.7%)	966 (100.0%)	

*SD* standard deviation, *HR* hormonal receptor, *TNBC* triple negative breast cancer.

## Discussion

In this study, we performed high-throughput proteomics analysis on the FFPE samples of BCs with varying times between initial surgery to metastatic event. We used a machine learning-based feature selection scheme on the proteomics data, and implemented MI and NP to narrow down into the potentially meaningful biomarkers regulating metastatic potential.

Since the introduction of high-throughput profiling technologies, enormous expression data on transcriptomics and proteomics levels has been accumulated. Differential expression analysis or overrepresentation analysis are commonly used to identify biomarkers of interest within the dataset; however, it is often very difficult to interpret the influence of the markers within the context of biological network. In contrast, NP can simulate the effects of biomarkers in a biological network by propagating the influence of the candidate molecule via protein–protein interactions (PPI) analysis.

By using this method, we identified and found VWA5A as the most valuable marker of metastatic potential of BC. *VWA5A* gene, also known as *BCSC1* or *LOH11CR2A*, is located within the chromosome 11q23–q24, which has been known to be frequently (ranging from 45 to 63%) deleted in various cancers including breast, ovary, uterine cervix, and lung^11–15^. Within the genomic loci, *VWA5A* gene was first cloned in 1997, and the subsequent functional study suggested that *VWA5A* acts as a tumor suppressor gene^16,17^, where overexpression of *VWA5A* in MCF7 cell line resulted in enhanced tumorigenicity^16^. However, interrogation of the clinical implication of VWA5A in BC has not been thoroughly performed.

In our proteomics discovery data, the expression of VWA5A gradually decreased as the clinical features of each group worsened, from NM, LM, and finally M groups, which was in line with previous studies that designated VWA5A as a tumor suppressor^18^. Additional biological network-based feature selection also suggested this molecule as the reliable biomarker representing metastasis-prone BCs. Notably, our discovery set was composed of variable subtypes of BCs, though HR+HER2− type dominated. Strong association of VWA5A in this heterogeneous discovery population and metastasis-related clinical behavior prompted us to hypothesize that the assessment of VWA5A protein expression could be used as the initial screening method for prediction of poor prognosis, regardless of the BC subtype.

To gain more evidence on the clinical implication of VWA5A, we performed a survival analysis using the publicly available METABRIC dataset. Though significant differences in DFS and OS were not ascertained in HER2+ and TNBC subsets, a strong association was found in the HR+ group. Nevertheless, additional in vitro experiments on HR+ and TNBC cell lines using siRNA showed that VWA5A loss resulted in a marked increased in invasiveness and migratory potential of these cells, suggesting that VWA5A still matters at least in HR+ and TNBC subtypes of BCs. The limitations of our in vitro experimental design include lack of VWA5A overexpression assay, rescue experiments of VWA5A, and in vivo functional validation of VWA5A. However, consistent consequences of VWA5A knockdown across multiple types of BC cell lines cautiously support the tumor-suppressive nature of the biomarker.

The pervasive role of VWA5A in suppressing variable types of BCs may result from the common cellular mechanism associated with metastatic behavior. *VWA5A* lies in the vicinity of *CHEK1*, *PIG8*, and *ROBO3* loci, and this genomic region is known to be commonly deleted together^19^. Therefore, *CHEK1* deletion coupled with VWA5A loss would result in impaired DNA damage response, contributing to genomic instability and resultant aggressive behavior of the BC cells^20–22^. Another possible explanation would include the interaction between tumor cells and extracellular matrix. It is reported that the tissue expression of VWA5A showed an inverse correlation with the expression of MMP14^23^, a form of matrix metalloproteinase (MMPs). MMPs play pivotal roles in tumor invasion and metastasis by participating in ECM degradation and activating other MMP family members in various types of solid tumors^24–27^. In addition, it would be intriguing to investigate whether the loss of VWA5A causes tumor cell-intrinsic alterations—i.e. epithelial mesenchymal transition (EMT)—along with ECM modulation, as suggested by previous reports^28–31^.

In the area of breast cancer research, recent advances were developed mostly using cutting-edge technologies, and it is often very difficult to robustly implement the findings into routine clinical practice. However, we found that expression of VWA5A can readily be performed by IHC on archival FFPE tissues. Nuclear expression of VWA5A on the surgically resected BC tumor cells could easily be assessed by the pathologist semi-quantitatively, and as the expression levels decreased, significant correlations with advanced T stage and the presence of lymphatic invasion were noted in the validation cohort composed of consecutive BCs of all subtypes. Pathological T stages and lymphatic invasion status were factors retrieved from the pathology reports of surgically resected BCs, which means that these features can seldom be assessed in the limited, needle biopsy specimens. Therefore, assessment of VWA5A IHC on the initial diagnostic needle biopsy specimen of BCs of all types can provide not only prognostic information but also clinically meaningful adjunctive information, especially the ones that cannot be readily assessed in biopsy samples.

The lack of significant association between VWA5A IHC levels and survival may stem from the intrinsic limitation of the validation cohort—the population was composed of consecutive patients with surgically resected BCs, thus only a limited number of patients developed distant metastasis in these operable cases. Therefore, future studies using the initial breast biopsy samples of metastatic BCs would be able to further ascertain the performance of VWA5A expression on predicting the metastatic regulatory potential.

Another limitation of our study is the relative underrepresentation of HER2+ BCs. HER2+ BCs accounted for 13.8% of the patients in the discovery set and 15.8% in the validation set, which were less than the TNBC subtype. In addition, the lack of survival correlation between *VWA5A* gene expression and survival data according to METABRIC analysis implies that the biological role of VWA5A may be different in HER2+ BCs unlike HR+ BCs or TNBCs. HER2+ BCs are notably characterized by aggressive behavior including distant metastasis, despite advances in targeted therapies. Especially, HER2+ BCs show a propensity toward brain metastasis, and accurate prediction of this aggressive phenomenon is one of the most important unmet needs in the management of HER2+ BC. Therefore, the discovery of biomarkers for metastasis prediction and prognostication in HER2+ BC should be performed in the near future by implementing a multi-omics approach.

In summary, we discovered lower expression of VWA5A as the potential biomarker for metastasis-prone BCs by high-throughput proteomics assay followed by machine learning-based protein expression analysis and feature selection. In addition to the prognostic significance assessed by METABRIC cohort analysis, the biological influence of VWA5A knock-out confirmed the tumor suppressive role of VWA5A. We suggest the clinical utility of VWA5A IHC, where further validation of VWA5A IHC adjunctive tools for prognostication should focus on the large-scale verification of the population with higher numbers of distant metastasis and HER2+ BCs.

## Methods

### Patient selection

In the discovery set for high-throughput proteomics analysis, 29 patients with BC diagnosed between 1998 and 2014 at Seoul National University Hospital (SNUH) with available clinical follow-up data including DFS, MFS, and OS were included. We defined the LM as the development of metastasis after 5 years from the diagnosis.

In addition, a retrospective consecutive cohort of 1002 patients with BCs who underwent surgical resection and were diagnosed between 2009 and 2012 in SNUH were recruited. Patients who received neoadjuvant chemotherapy were excluded. FFPE surgical samples archived in the Department of Pathology, SNUH, TMA were constructed. The experienced breast pathologist (H.S.R) selected the 2 mm-sized representative areas from H&E slides of the samples, and the TMAs were made (Superbiochips Laboratory, Seoul, Republic of Korea).

This study was approved by the Institutional Review Board (IRB) of SNUH, and the individual informed consent forms from the patients were waived by the decision of IRB. We confirm that all methods were performed in accordance with the relevant guidelines and regulations.

### High-throughput proteomics assay

From the FFPE blocks of surgically resected samples of the discovery set, 10 μm thick sections were deparaffinized in xylene twice. The protein uses a combination of acetone precipitation and filter-aided sample preparation (FASP)^32^. Desalted pooled peptides were fractionated using the stage tip-based high-pH peptide fractionation method^32^.

The pre-fractionated peptides were analyzed on an LC–MS system with an Easy-nLC 1000 (Thermo Fisher Scientific, Waltham, MA, USA) equipped with a nanoelectrospray ion source (Thermo Fisher Scientific) and Q-Exactive mass spectrometer (Thermo Fisher Scientific). The peptide samples were separated into a trap column and an analytical column (Thermo Fisher Scientific).

Raw MS/MS files were processed with MaxQuant (version 1.6.1.0) using the Andromeda search engine against the Human Uniprot protein sequence database (December_2014, 88 657 entries). Experiment details are presented in the Supplementary Methods.

### Proteomics data analysis

#### Preprocessing

Protein levels were normalized and statistical analyses were performed by Perseus (version 1.5.8.5). ANOVA was used to determine DEP among NM, LM, and M groups. Peptide intensity data from tandem mass spectrometry (MS/MS) were processed using MaxQuant acquired. LFQ intensity was used as a protein quantification measure. The proteome dataset of breast cancer metastasis was preprocessed as follows. Only proteins that were identified in all replicates were used. Missing values were predicted by k-nearest neighbor imputation. As a result, in total 9455 proteins were quantified and resulted in 6639 proteins after filtration and imputation. The MS-based proteomics data of all identified peptides has been registered in the ProteomeXchange Consortium (http://proteomecentral.proteomexchange.org) via the PRIDE partner repository (data set identifier: PXD015171).

#### Machine learning-based feature selection

The goal is to select proteins as features for biomarker candidates whose abundance level can discriminate metastasis types: NM, M, and LM. The sample size of each metastasis type is 9 for NM, 10 for M, and 10 for LM, respectively.

**Figure 5:**
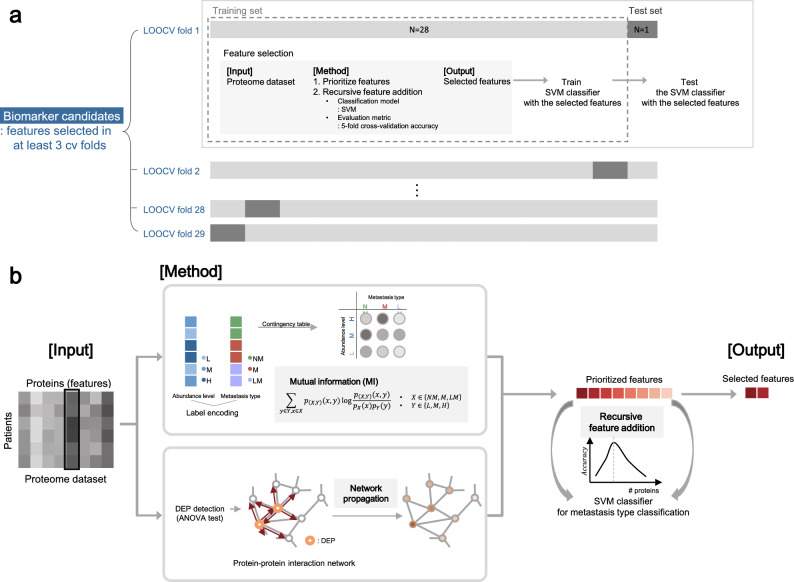
Schematic overview of biomarker candidate selection. (**A**) Leave-one-out cross-validation (LOOCV) scheme for biomarker candidate selection. For each CV fold, a sample was used as a held-out test set, while the other samples were used for feature selection. After feature selection, SVM classifier was trained with the selected features and tested with the test set. (**B**) Within each LOOCV training set, we prioritized proteins by mutual information (MI) network propagation (NP). Proteins were ranked in terms of the discriminative power of metastasis type measured by MI. After feature prioritization, features were added to the classification model via recursive feature selection, until the accuracy reached the maximum.

The full schematic overview of the biomarker candidate selection method is illustrated in Fig. 5 and the pseudo-code of the algorithm is in Supplementary Fig. S6. Our feature selection method operates in two hierarchical levels. The higher level is to choose protein features that are selected multiple times in the 29 LOOCV experiments. On the lower level of each of 29 LOOCV experiments, a set of features is selected in the recursive feature addition scheme. Below we explain how the algorithm works.

At a higher level (Fig. 5A, Supplementary Fig. S6; line 2–5, 23–25), we used the LOOCV scheme for biomarker candidate selection to identify protein sets whose abundance can discriminate metastasis type of unseen patients. For each CV fold, a sample was used as a held-out test set (Supplementary Fig. S6; line 4), while the other samples were used as a training set for feature selection (Supplementary Fig. S6; line 5), denoted as *tr_LOOCV*. In each of 29 LOOCV folds, we performed feature selection by recursive feature addition and metastasis prediction tasks in fivefold CV settings. To evaluate the selected features, SVM classifier was trained with the selected features and tested with the test set in terms of precision, recall, and F1 score. We reported macro averaged evaluation metrics of precision, recall and F1 score respectively, which is to calculate an evaluation metric for all classes individually and average them. As a result, a different feature set is selected and tested for each of 29 LOOCV folds. Thus, the final features are features that are selected multiple times, 3 times, out of 29 LOOCV folds. Since we are selecting features out of 6639 protein features that are measured by mass spectrometry experiments, this is a very stringent feature selection criteria. Statistically, we can measure p-values for features selected as features at least 3 times as $1.95\times {10}^{-6}$.

At the lower level (Fig. 5B), in each of the 29 LOOCV folds, protein features were selected by performing recursive feature addition (Supplementary Fig. S6; line 6–22). The first step is to prioritize proteins for the recursive feature addition analysis using all samples in *tr_LOOCV* (Supplementary Fig. S6; line 6). Proteins were ranked in terms of the discriminative power of metastasis type measured by MI. When there were ties for multiple proteins, we used the NP method to break the ties. The second step is to select features by recursive feature addition (Supplementary Fig. S6; line 7–22). Specifically, protein features were recursively added to an SVM classifier according to the rank until the prediction performance of the classifier reached peak. The prediction performance is measured with fivefold cross-validation within a *tr_LOOCV* (Supplementary Fig. S6; line 10–17). The feature set where prediction performance is maximum was regarded as the final selected feature for each LOOCV fold (Supplementary Fig. S6; line 18–21).

##### Feature prioritization step

MI is a metric for statistical dependency between two variables, the metastasis label and protein abundance group in this case. Relevance between protein abundance level and metastasis type is computed as below.

$$\sum_{y\in Y}\sum_{x\in X}{p}_{\left(X,Y\right)}\left(x,y\right){\text{log}}\frac{{p}_{\left(X,Y\right)}\left(x,y\right)}{{p}_{X}\left(x\right){p}_{Y}\left(y\right)}.$$

Discrete variable $X\in \{NM,M,LM\}$ denotes metastasis type and the other discrete variable $Y\in \{L,M,H\}$ denotes protein abundance level, while $L$ encodes for low abundance level, $M$ for medium abundance level, and $H$ for high abundance level. Since raw LFQ protein abundance is a continuous variable, we transformed the variable into a multi-chotomous discrete variable $X$ where protein abundance levels are categorized into 3 groups, where $L$ for low, $M$ for medium, and $H$ for high abundance level, respectively. To handle the label imbalance issue, we sampled 100 times with an equivalent sample size (n = 9) for each metastasis type. Averaged MI across sampling batches was used to prioritize proteins.

To prioritize features in terms of influence in a biological network, NP simulates the effects of biomarkers in a biological network. In detail, NP re-prioritized proteins according to the relevance with the seed proteins, by propagating the influence of seed proteins along the PPI given a STRING (v11.0)^33^ as a protein–protein interaction network. For seed selection, DEPs among the three metastasis types were detected with the ANOVA test, for proteins with adjusted p-value < 0.1 after Benjamini–Hochberg multiple test correction. For a network, STRING network was trimmed with a confidence score, where normalized edge confidence < 0.5 was filtered out. For the implementation of the network propagation algorithm, we used HotNet2^34^.

##### Feature selection step

After feature prioritization, features were recursively added to the classification model according to the feature rank. For evaluating each iteration of the recursive feature addition, SVM with an RBF kernel was trained to discriminate the metastasis types by recursively adding features according to the rank. The SVM classifier with RBF kernel was chosen as a classifier because the model showed the highest prediction performance compared to the other classifiers (Supplementary Table S1). As a new feature was added, the prediction performance, i.e., accuracy, of the current classification model in each iteration was computed in a threefold cross-validation scheme with a training set. Accuracy is calculated as macro averaged accuracy for multiclass classification^35^. Feature addition was iteratively performed until the accuracy reached the maximum.

### Cross validation using publicly available gene expression dataset

To ascertain the validity of the candidate biomarker derived from the proteomics assay, we used the publicly available gene expression dataset—METABRIC^36^—to assess the prognostic significance of biomarker expression. Kaplan–Meier survival analyses according to *VWA5A* gene expression level was done via web-based analytic platform, CTGS (http://ctgs.biohackers.net/)^37^ accessed at June 2020.

### Cell culture and invasion/migration assay

We used HR+ cell lines (T47D, MCF7), HER2+ cell lines (HCC1954, JIMT-1, and SKBR3), TNBC cell lines (BT20, HCC70, HCC1143, HCC1937, MDA-MB-468, MDA-MB-231 and HCC 1395) for in vitro experiments. All cell lines were obtained from the Korean Cell Line Bank (KCLB, Seoul, Republic of Korea). T47D and HCC70 were cultured in RPMI (Gibco, Carlsbad, CA, USA), and BT20 was cultured in DMEM (Gibco) containing 10% fetal bovine serum (FBS; Gibco) and 1% penicillin and streptomycin (Gibco). The cells were maintained at 37 °C in a humidified atmosphere of 95% air and 5% CO_2_ and were screened periodically for *mycoplasma* contamination.

siRNAs targeting *VWA5A* were generated and purchased from Bioneer (Daejeon, Republic of Korea). T47D, BT20 and HCC700 cells were transfected with siVWA5A using Lipofectamine RNAiMAX (Invitrogen) according to the manufacturer’s instructions. After an incubation period of 48 h, mRNA expressions of each biomarker were assessed by RT-PCR and compared with the controls. RNA from the cells was isolated using TRIzol (Invitrogen), and synthesized respective cDNA was amplified using specific primers and HIPI plus Master mix (ElpisBio, Daejeon, Republic of Korea).

Cell migration and invasion assays were performed using 24-well inserts (Corning Incorporated, NY, USA) with 8-μm pores according to the manufacturer’s instructions. For transwell migration assays, 5 × 10^5^ cells were inoculated into the upper chamber, while culture medium containing 10% FBS was added into the lower chamber. After 24 h of incubation, the cells on the top of the membrane were removed, and the migrant cells were washed with phosphate-buffered saline (PBS), stained by 1% crystal violet for 10 min, and counted in 3 randomly selected fields under the microscope (Nikon, Tokyo, Japan). The experiments were replicated three times each.

For Matrigel invasion assay, the upper wells of Boyden chambers (Corning) were coated with 2 mg/ml of Matrigel at 37 °C incubator with 5% CO_2_. 5 × 10^5^ cells were put into the upper chamber, with 10% FBS added medium in the lower chamber. Invaded cells, after 24 h of incubation, were processed and counted in triplicate, as described above.

### Analysis of cell proliferation

To measure cell proliferation rate depending on VWA5A gene expression, cells were seeded at 1 × 10^4^ cells per well in a 96-well plate. Cell proliferation was determined using the Cell Counting Kit-8 (Dojindo Laboratoried, Japan) every day for 3 days.

### Immunoblotting

Whole proteins of breast cell lines were extracted using T-PER (Pierce, Rockford, IL) containing a cocktail of protease inhibitors (Roche). Proteins were detected using standard immunoblotting procedures and the appropriate primary antibodies. Anti-BCSC was purchased from Novus Biologicals (Centinnial, CO) and anti-β-actin was purchased from Santa Cruz Biotechnology (Dallas, TX).

### qPCR

Cultured cells were lyzed in Trizol (Takara, Japan) and total intracellular RNAs were extracted according to the manufacturer’s instructions. cDNA was generated using M-MLV reverse transcriptase (Promega, Madison, WI) and random primers (Promega). Relative quantitative real-time PCR was performed using GO Taq® qPCR Master Mix (Promega) and analyzed on a CFX96™ Real-Time System (Bio-Rad, Hercules, CA).

### Immunohistochemistry

From the TMA block of the validation set, 4 μm thick sections were taken to perform IHC for VWA5A. Monoclonal anti-VWA5A antibody (dilution 1:400; clone OTI3D6; Novus Biologicals, Centennial, CO, USA) was used, and immunostaining was performed using Benchmark automatic immunostaining device (Ventana BenchMark XT Staining System, Tucson, AZ) following the manufacturer’s guidelines. Interpretation of the IHC slides were done by a breast pathologist (K.J.) using histoscore (H-score) while blinded to the clinicopathological characteristics. H-score of 50 was used as a cut-off value discriminating VWA5A-high and VWA5A-low.

### Statistical analysis

We used the chi-square test and linear-by-linear tests to compare categorical variables and the Kruskal–Wallis test to compare continuous variables, as appropriate. Kaplan–Meier survival analyses were performed based on the log-rank method. Harrell’s c-index was used to assess the discriminating ability of biomarker expression. Statistical significance was defined as the *p*-value less than 0.05. All analyses were performed by SPSS software (version 25; IBM SPSS Statistics, IBM Corporation, Armonk, NY, USA) and R version 3.6.3 (www.r-project.org).

### Ethics declarations and informed consent statement

This study was approved by the Institutional Review Board (IRB) of SNUH (IRB No. 1612-011-811), and the individual consent forms from the patients were waived by the decision of IRB.

### Supplementary Information

Supplementary Information.

## Data Availability

The datasets generated during and/or analyzed during the current study are available from the corresponding author on reasonable request.
