# Supplementary material for: Identification of VWA5A as a novel biomarker for inhibiting metastasis in breast cancer by machine-learning based protein prioritization
Source: Sci Rep. 2024 Jan 30;14:2459. doi: 10.1038/s41598-024-53015-1 (PMC10828438; doi:10.1038/s41598-024-53015-1)
Supplement: Supplementary file 1 — Supplementary Information. [file 41598_2024_53015_MOESM1_ESM.pdf]

Supplementary Information

Identification of VWA5A as a Novel Biomarker for Inhibiting Metastasis in Breast Cancer by Machine-learning Based Protein Prioritization

Jiwon Koh[1 †], Dabin Jeong[2 †], Soo Young Park[3†], Dohyun Han[4,5], Da Sol Kim[3], Ha Yeon Kim[3], Hyeyoon Kim[4], Sohyeon Yang[1], Sun Kim[2,6*], Han Suk Ryu[1,3,7,8*]

[1]Department of Pathology, Seoul National University Hospital, Seoul National University College of Medicine, Seoul, South Korea.

[2]Interdisciplinary Program in Bioinformatics, Seoul National University, Seoul, South Korea.

[3]Department of Pathology, Seoul National University College of Medicine, Seoul, Republic of Korea.

[4]Proteomics Core Facility, Biomedical Research Institute, Seoul National University Hospital, Seoul, South Korea

[5]Transdisciplinary Department of Medicine & Advanced Technology, Seoul National University Hospital, Seoul, South Korea

[6]Department of Computer Science and Engineering, Institute of Engineering Research, Seoul National University, Seoul, South Korea

[7]Cancer Research Institute, Seoul National University, Seoul, Republic of Korea

[8]Pharmonoid Co., Ltd., Seoul, Republic of Korea

†Jiwon Koh, Dabin Jeong and Soo Young Park contributed equally as main authors.

[*]Sun Kim and Han Suk Ryu jointly supervised this work.

Supplementary Methods

Protein Extraction and Peptide desalting

FFPE tissues were sectioned 10-µm using microtome. The slide of each samples were stained with hematoxylin and eosin and mounted under a glass coverslip. Using the H&E slides, only tumor tissues were selectively displayed through visual analysis by pathologists.

The FFPE slides were deparaffination xylene for 5min and 3min. Samples were then hydrated in 100% ethanol for 90 seconds, followed by 75% ethanol for 90 seconds, and distilled water for 90 seconds. The extracted tissues were lysed in 4% SDS, 2 mM TCEP, and 0.1 M tris-HCl, pH 7.5 buffer followed by direct sonication (10%, 5 cycles, 5 s, 2 s). Lysates were heated for 2hr at 95 °C. After 10min centrifugation (15,000rpm, 21°C), the concentration of protein was measured using a reducing agent-compatible BCA assay (Thermo Fisher Scientific, Waltham, MA). Then, each sample containing 200 µg of total protein was precipitated with cold acetone overnight.

The pellet was resuspended in denaturation buffer containing 2% sodium dodecyl sulfate (SDS), 10 mM tris(2-carboxyethyl) phosphine (TCEP), 50 mM chloroacetamide (CAA), and 0.1 M tris-HCl, pH 8.5 and heated for 15 min at 95 °C. 300µL of UA buffer (8 M UREA in 0.1 M Tris-Cl, pH 8.5) were added to sample and the mixtures were loaded onto 30-kDa spin filter (Merck Millipore, Darmstadt, Germany). The 20ug of eluted peptides were acidified with trifluoroacetic acid (TFA). Peptides were than desalted using StageTip-C18 as follows. Dried peptides were resolved with loading solution (15mM ammonium hydroxide and 2% acetonitrile) and separated with StageTip based microcolumn prepared as described in the protocol (DOI: 10.1039/C9AY01269A). The fractionated peptides were lyophilized for LC-MS analysis.

Mass spectrometry and Database Search

The pre-fractionated peptides were analyzed on an LC–MS system with an Easy-nLC 1000 (Thermo Fisher Scientific, Waltham, MA, USA) equipped with a nanoelectrospray ion source (Thermo Fisher Scientific) and Q-Exactive mass spectrometer (Thermo Fisher Scientific). Peptide samples were separated on a two-column system. The maximum ion injection times for the full scan and MS/MS scan were 20 and 100 ms, respectively. Raw MS/MS files were processed with MaxQuant (version 1.6.1.0) using the Andromeda search engine against the Human Uniprot protein sequence database (December_2014, 88 657 entries). Primary searches were performed using the MS/MS ion tolerance and set to 20 ppm. Cysteine carbamidomethylation N-acetylation of protein and oxidation of methionine were set as fixed and variable modifications, respectively. Enzyme specificity was set to full tryptic digestion. The required false discovery rate (FDR) was set to 1% at the peptide, protein, and modification level. We enabled the ‘Match between Runs’ option on the MaxQuant platform to maximize the number of quantification events across samples

Supplementary Table Legends

Supplementary Table 1. Prediction performance support vector machine (SVM) classifier.

	Without feature selection			Recursive feature addition		
	Precision	Recall	F1 score	Precision	Recall	F1 score
Logistic regression	0.38	0.53	0.44	0.55	0.48	0.48
Random Forest	0.48	0.51	0.49	0.49	0.48	0.48
SVM (rbf)	0	0	0	0.8	0.79	0.79

Supplementary Table 2. Differential expression of 9 candidate biomarkers

Protein	VWA5A	TUBB	RPL21	ZNF112	HSPA12B	ERLIN1	POLR2L	ALOX5	RPL5
LM_1	27.98844	36.29002	29.31693	24.42544	28.92325	26.27857	26.2756	28.62373	28.12406
LM_2	28.88043	36.07484	27.0345	25.6295	28.44446	26.52453	24.83638	28.39975	26.9267
LM_3	29.37441	36.0825	28.87523	25.0518	28.89326	27.90327	24.63951	28.55046	27.34983
LM_4	30.3023	36.05976	29.79909	25.27546	28.92848	26.81275	25.80636	28.72864	27.14812
LM_5	29.18922	36.23763	28.59489	23.7525	28.51335	26.50406	24.61275	28.62552	26.96787
LM_6	28.57739	36.03761	28.99184	22.98454	29.07894	26.50038	24.3464	28.66321	27.67903
LM_7	28.68413	35.73208	26.92162	24.68415	28.19288	26.3844	24.31514	28.43585	27.20411
LM_8	29.24601	36.01704	28.31684	24.03635	28.18177	25.85006	25.17086	28.38892	28.17255
LM_10	30.29495	36.54166	28.02841	24.64466	28.44028	26.49219	24.91955	28.13736	26.76662
M_1	27.23441	36.61496	29.58596	22.18785	27.90249	26.13363	24.65993	28.2588	27.37449
M_2	28.49482	36.26598	28.89998	22.08497	27.82353	26.01626	24.7361	28.54134	25.86771
M_3	28.25378	36.34166	29.71757	22.02093	28.37118	26.73394	24.29914	27.30257	26.58747
M_4	27.72069	36.42664	31.20724	21.52416	26.84798	26.79806	24.84263	28.15965	27.7562
M_5	28.25401	36.85943	29.85239	22.57997	27.57862	26.77977	25.17679	26.97531	26.78049
M_6	29.09288	36.35409	30.13974	22.64206	27.54874	26.85119	23.23261	27.45485	27.31404
M_7	27.45017	36.33296	29.78088	22.94	27.73476	26.53755	24.40656	27.35772	27.28387
M_8	28.23887	36.61137	28.94469	22.18845	26.90938	26.87795	25.24458	28.25819	27.39976
M_9	29.34627	36.64477	29.26682	22.13395	27.05623	26.81426	25.68322	28.84116	27.24595

M_10	28.10071	36.40019	29.98992	23.31067	27.20584	26.28749	24.88031	27.70967	27.6839
NM_1	28.8756	35.86467	30.37842	24.12289	27.64128	27.19359	25.33952	29.62934	26.87499
NM_2	29.52201	36.29887	30.76815	21.77658	28.27673	26.45331	23.48506	29.22468	26.23608
NM_3	30.32979	35.79613	30.35623	24.13511	29.6567	27.39144	23.32986	29.28146	25.59728
NM_4	29.69957	36.00227	30.67431	23.37691	28.19358	27.14696	23.98025	29.11368	25.81577
NM_5	29.41444	36.34306	31.22588	23.84207	27.97392	27.00048	23.94412	29.79618	26.76585
NM_6	29.58787	36.13351	31.22116	23.89227	28.21636	27.01701	23.9213	28.30378	26.47656
NM_7	29.48333	35.92731	31.25347	23.30562	28.18167	26.88095	23.59096	28.335	25.38566
NM_8	29.78812	35.89918	30.5673	24.2559	28.79107	27.09753	23.79565	30.05011	26.53418
NM_9	30.05238	36.0127	27.80577	23.98997	28.06518	26.97284	23.89853	28.95523	25.85191
NM_10	29.93111	35.8716	31.63653	23.47422	29.50008	27.55769	23.16326	28.09224	25.73165
NM_median	30	36	31	24	28	27	24	29	26
NM_min	29	36	28	22	28	26	23	28	25
NM_max	30	36	32	24	30	28	25	30	27
LM_median	29	36	29	25	29	27	25	29	27
LM_min	28	36	27	23	28	26	24	28	27
LM_max	30	37	30	26	29	28	26	29	28
M_median	28	36	30	22	28	27	25	28	27
M_min	27	36	29	22	27	26	23	27	26
M_max	29	37	31	23	28	27	26	29	28

Supplementary Table 3. Association between pN stages and VWA5A expression within HR+/HER2- breast cancers in the validation set

		VWA5A-low	VWA5A-high	Total
pN stage	pN0	190 (57.8%)	162 (66.4%)	352 (61.4%)
	pN+	139 (42.2%)	82 (33.6%)	221 (38.6%)
Total		329 (57.4%)	244 (42.6%)	573 (100.0%)

Supplementary Fig. S1. Expression of VWA5A in the discovery set.

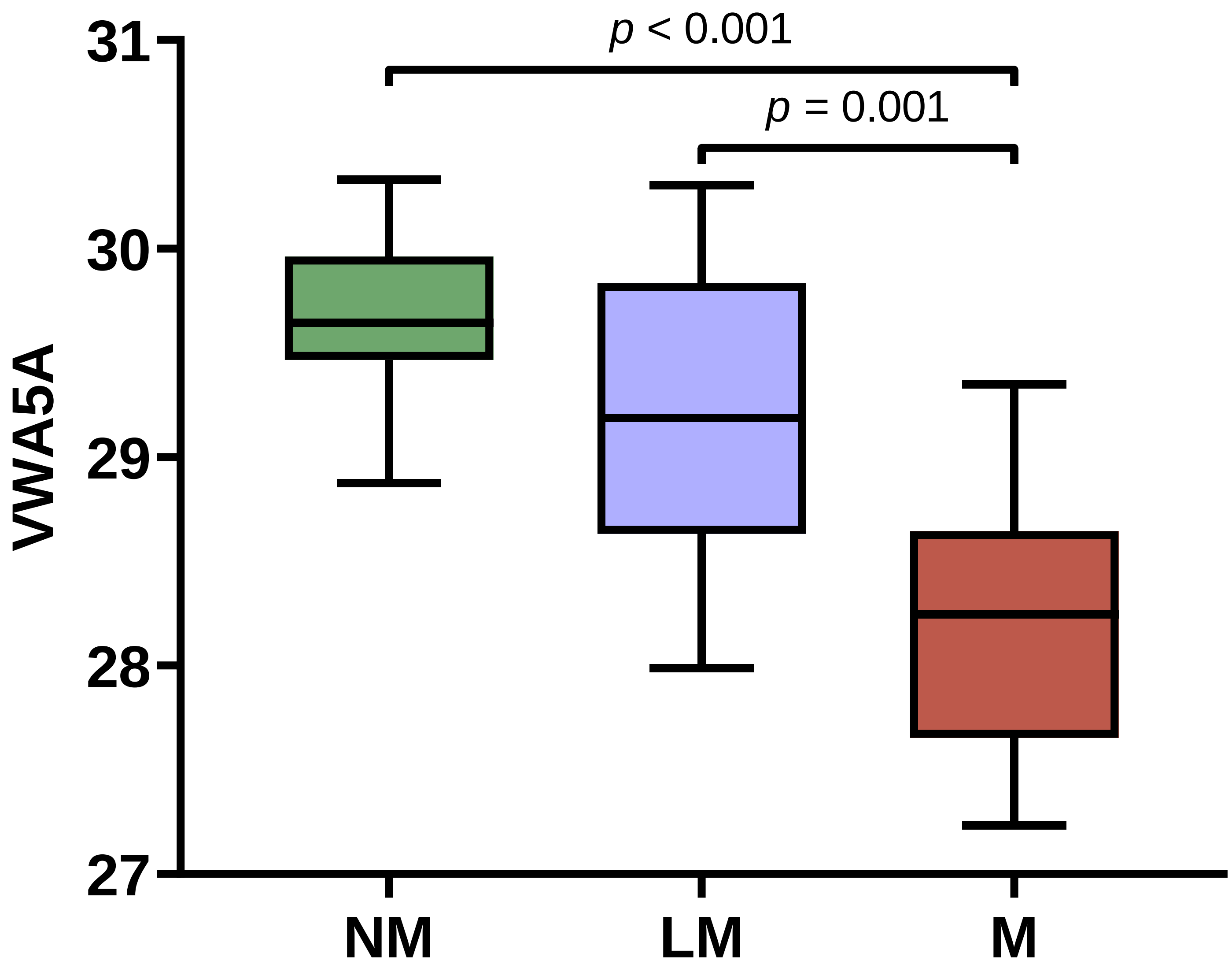

Supplementary Fig. S2. Confusion matrix depicting prediction performance with the selected features via recursive feature addition with leave-one-out cross-validation scheme.

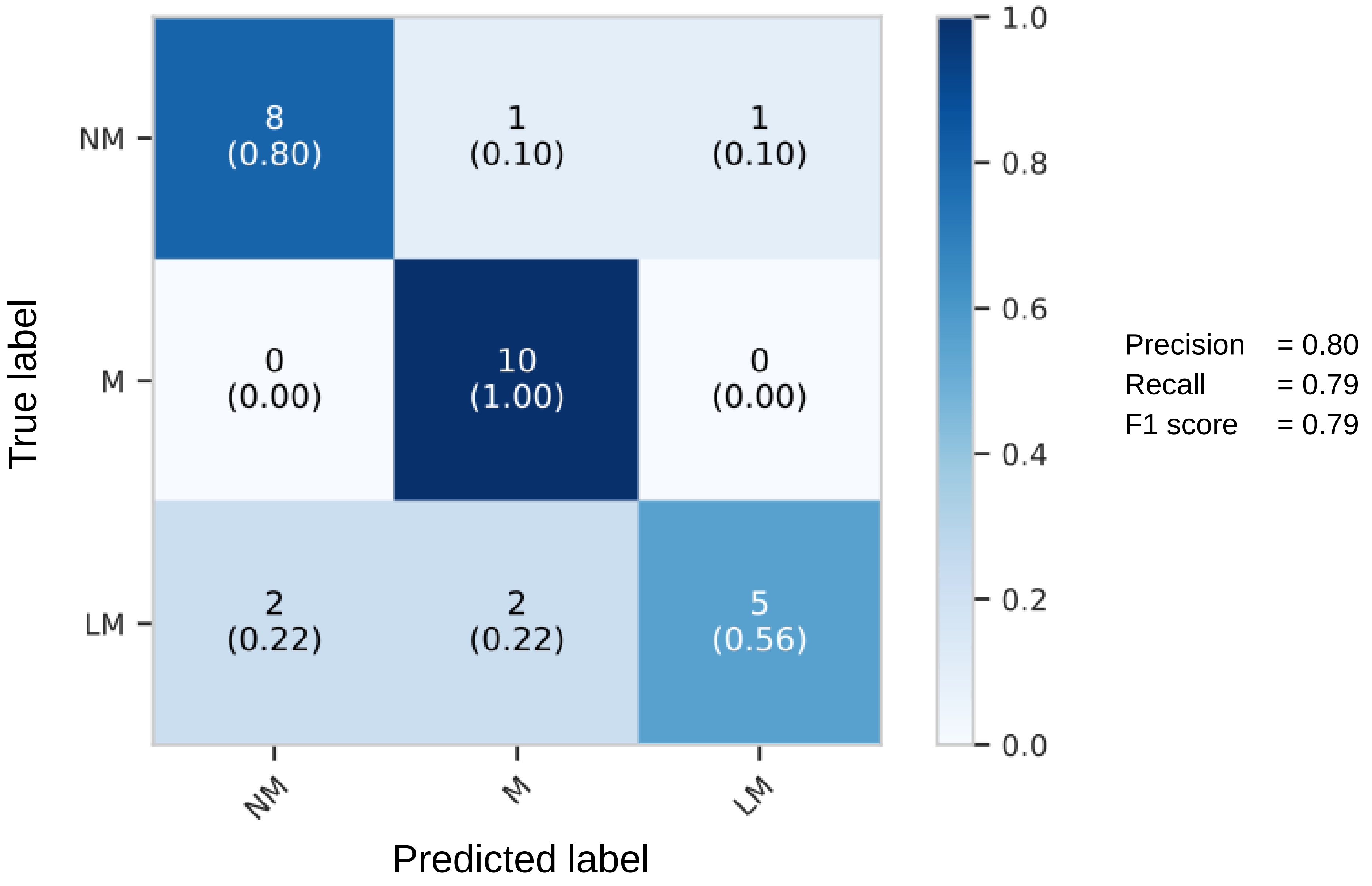

Supplementary Fig. S3. Mutual information and network propagation analyses

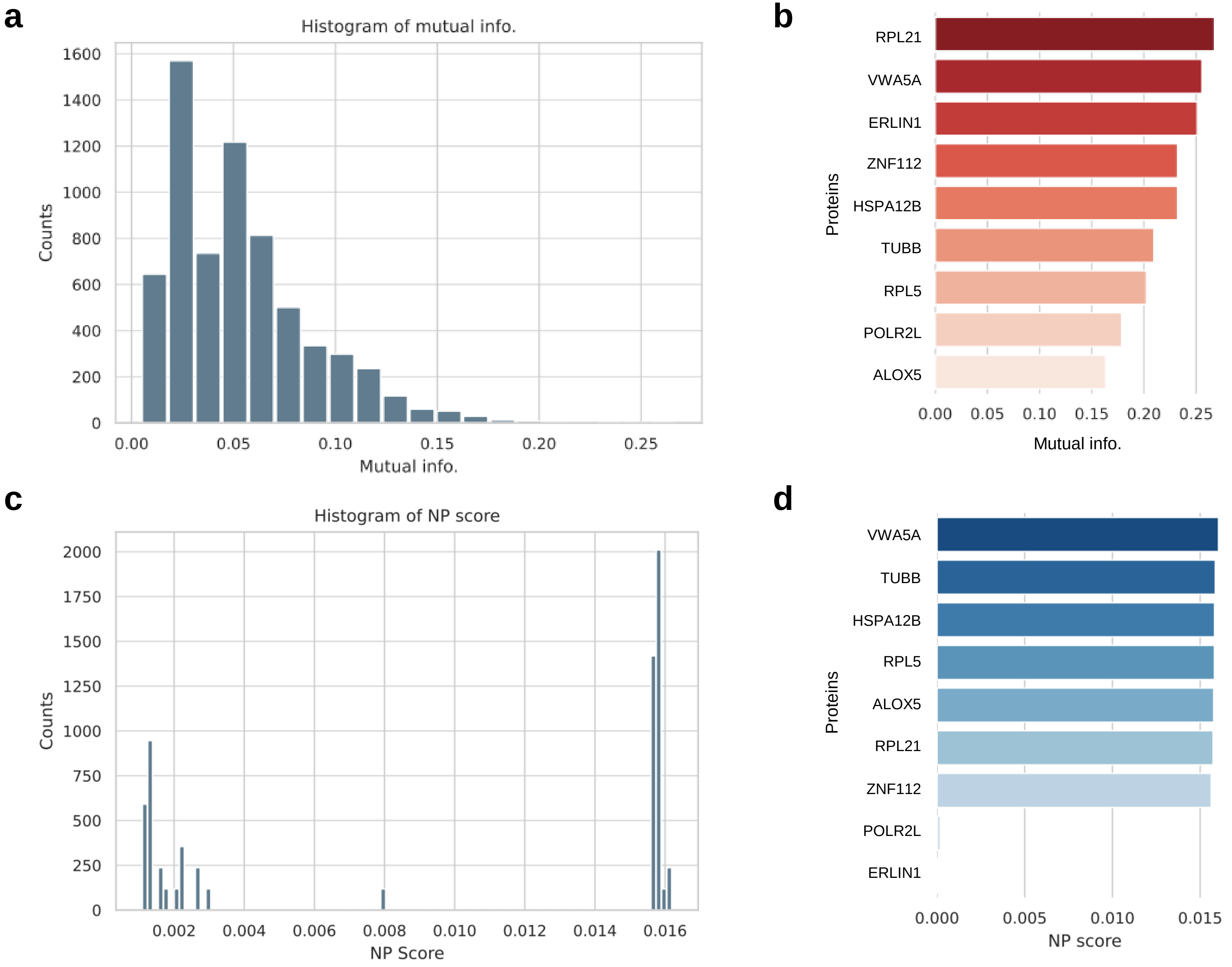

(a) Histogram of mutual information of 6,639 proteins from the whole patients. (b) Mutual information between metastasis types and protein abundance level for the nine biomarker candidates. (c) Histogram of network propagation (NP) scores of 6,639 proteins from the whole patients. (d) NP scores of the nine biomarker candidates.

Supplementary Fig. S4. Differences in overall survival according to the VWA5A expression levels in the pooled population and subtype-specific populations in METABRIC cohort.

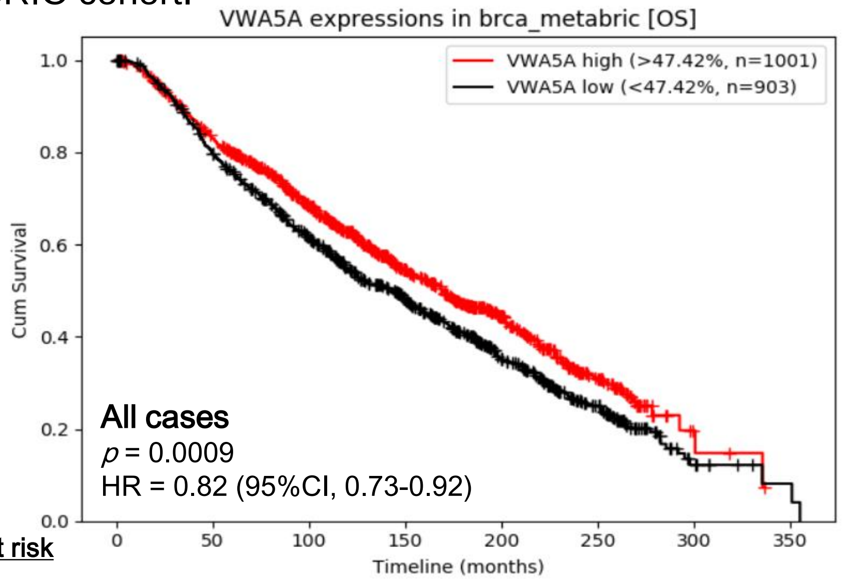

No. at risk

	0	50	100	150	200	250	300	350
VWA5A high	1001	835	702	599	543	499	489	487
VWA5A low	903	724	565	461	380	337	317	316

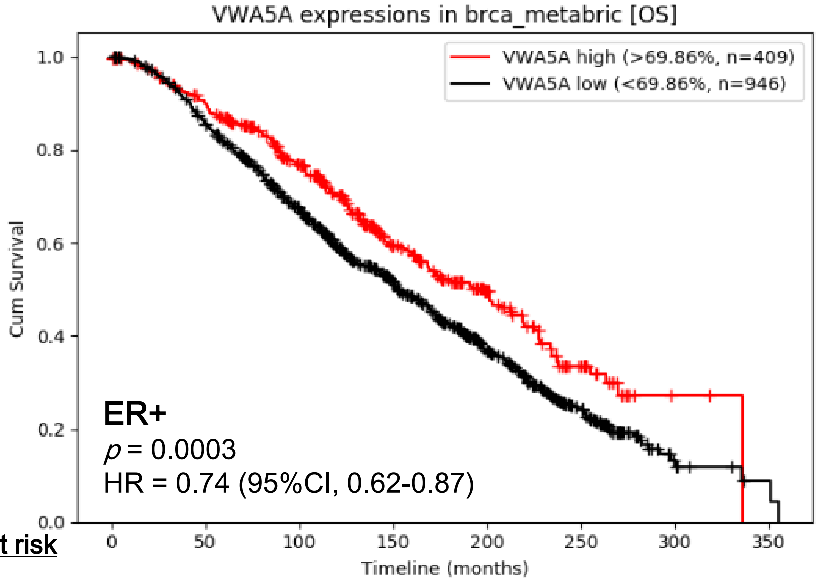

No. at risk

	0	50	100	150	200	250	300	350
VWA5A high	409	369	321	270	250	231	228	227
VWA5A low	946	812	644	520	423	365	345	343

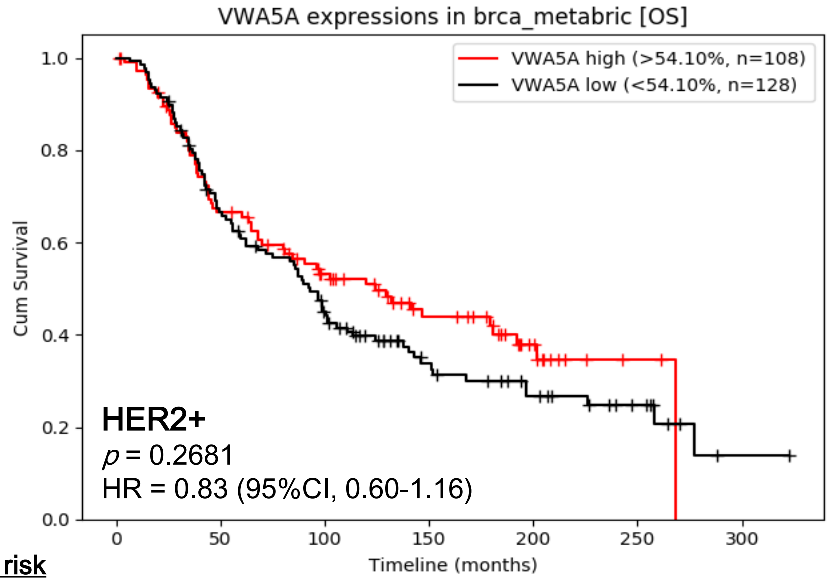

No. at risk

	0	50	100	150	200	250	300
VWA5A high	108	73	60	53	50	49	48
VWA5A low	128	86	60	49	44	43	41

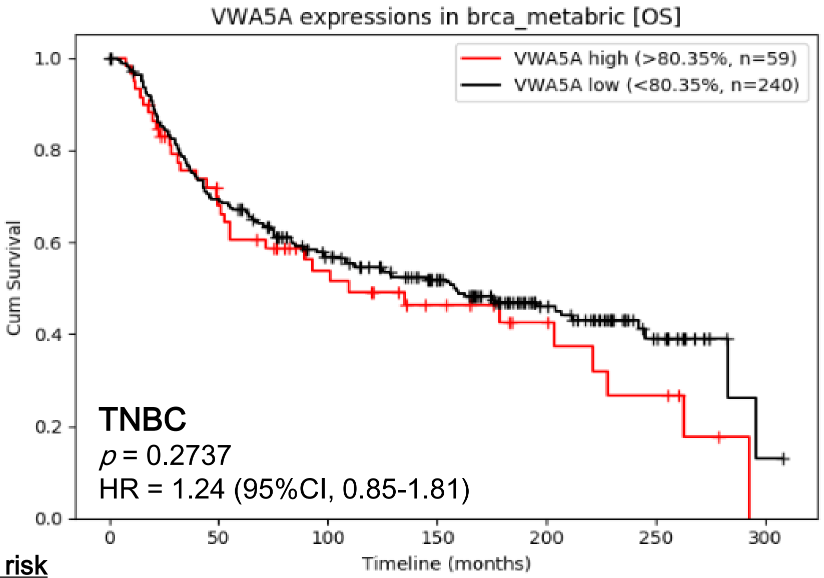

No. at risk

	0	50	100	150	200	250	300
VWA5A high	59	41	34	31	30	27	25
VWA5A low	240	167	139	129	120	115	113

Supplementary Fig. S5. Comparison of proliferation, mRNA and protein expression of the VWA5A gene according to subtypes of breast cancer cell lines.

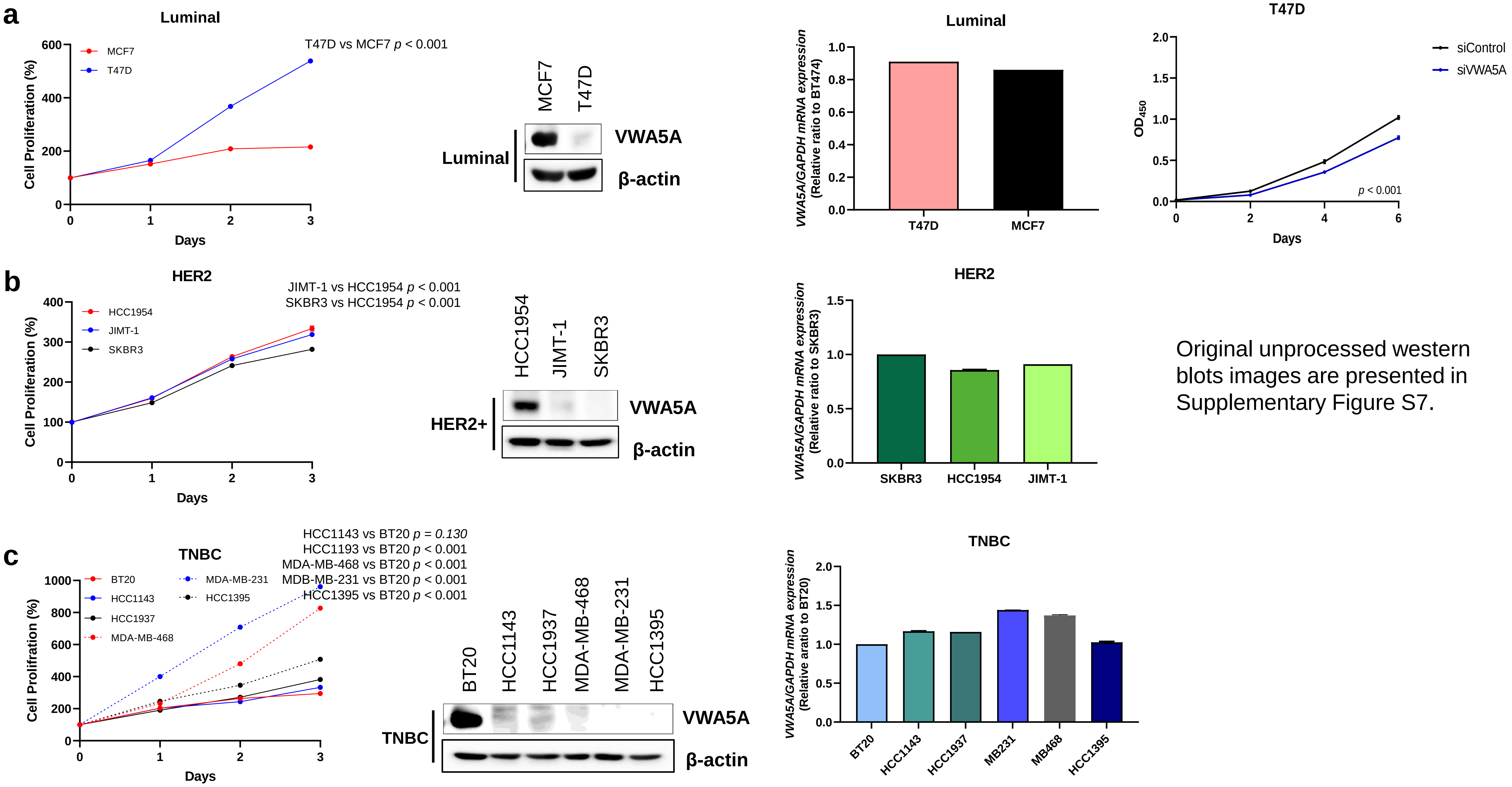

Original unprocessed western blots images are presented in Supplementary Figure S7.

Supplementary Fig. S6. Pseudo-code for biomarker identification algorithm.

Algorithm 1 Feature selection with recursive feature addition

Input D, dataset of N samples with M features $P = \{p_1, p_2, \cdots, p_M\}$
Output $P^\dagger$, a subset of P

procedure BIOMARKER IDENTIFICATION
    Divide data into $k_1, k_2, \cdots, k_N$ folds ▷ Leave-one-out cross-validation (LOOCV)
    for $i = 1$ to N **do**
        Set fold k_i as test set
        Set the remaining folds as train set (tr_LOOCV)
        Rank the feature set P using MI and NP score using tr_LOOCV ▷ Prioritize features
        $max\_performance = 0$, $P_i^* = \{\}$ ▷ Recursive feature addition
        for $j = 1$ to M **do**
            Set P_j as the top-ranked j features from the ranked P
            Split tr_LOOCV into $k_1^{'}, k_2^{'}, \cdots, k_5^{'}$ folds
            for $m = 1$ to 5 **do** ▷ Evaluate each feature addition
                Set fold $k_m^{'}$ as validation set
                Set remaining folds as train set (tr_5fold)
                Train SVM classifier with tr_5fold using P_j features
                Calculate $accuracy_m$ with $k_m^{'}$
            end for
            Calculate $accuracy$ as a mean of $accuracy_m$
            if accuracy $\geq max\_performance$ **then** ▷ Update selected feature set
                $max\_performance = accuracy$
                $P_i^* \leftarrow P_j$
            end if
        end for
        Calculate precision, recall, f1 score using P_i^* features with k_i test set ▷ Evaluate selected features
    end for
    return $P^\dagger$, a subset of P where each feature appears more than three P_i^*s

Supplementary Fig. S7. Original unprocessed western blot images.

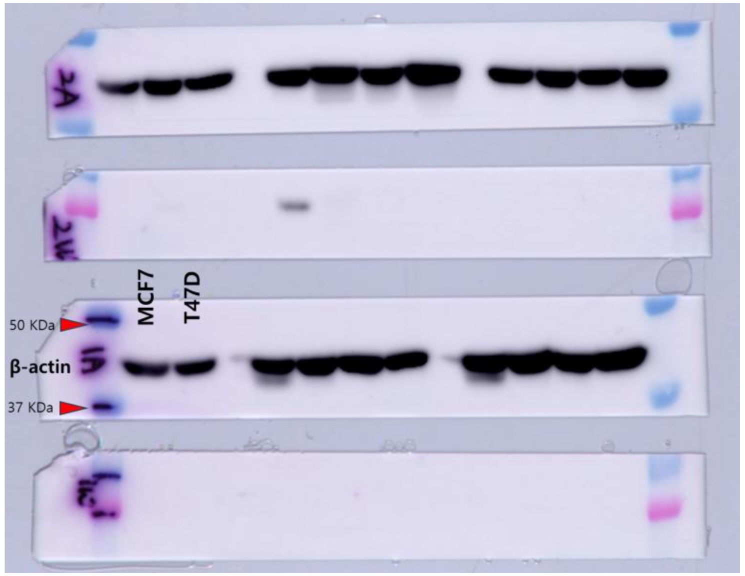

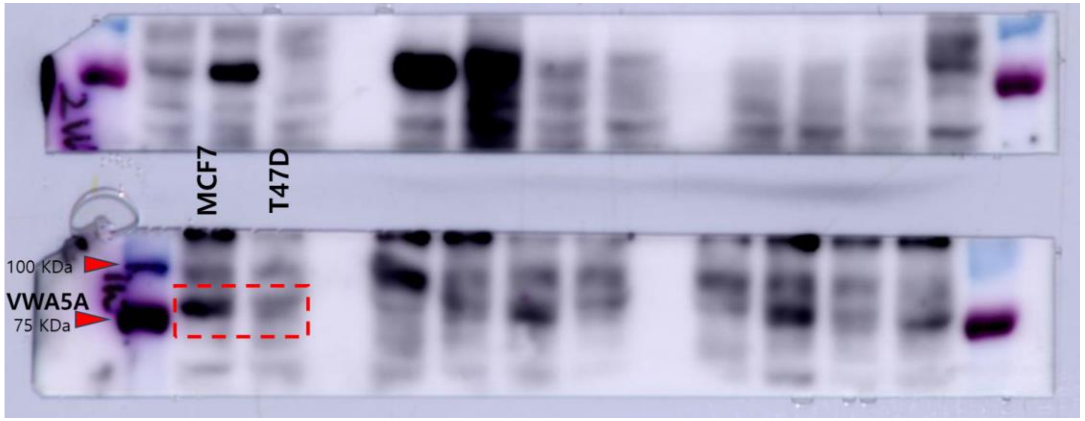

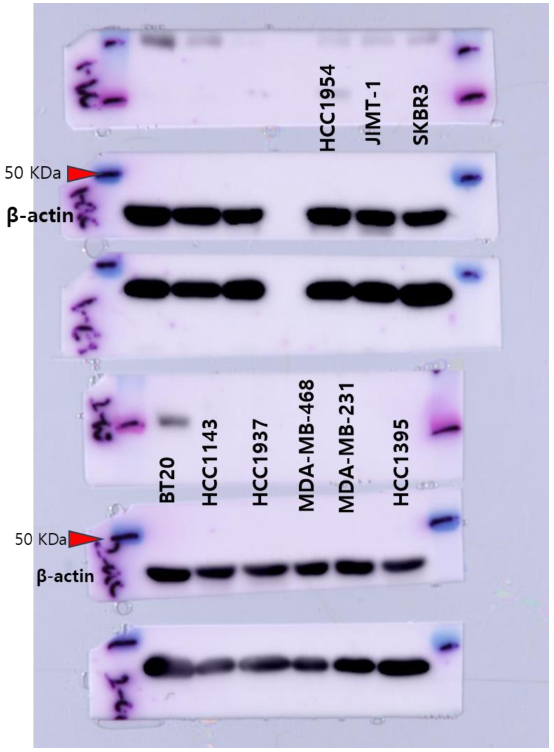

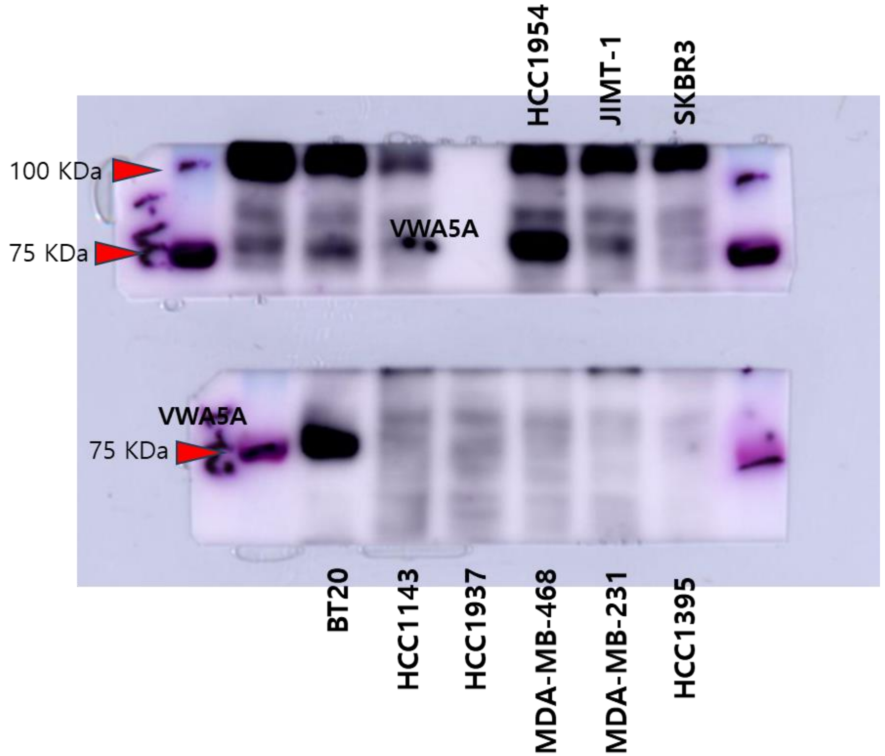

a. β-actin bands in two luminal type breast cancer cell lines, MCF7 and T47D. **b.** VWA5A bands in two luminal type breast cancer cell lines, MCF7 and T47D. **c.** β-actin bands in HER2+ breast cancer cell lines (HCC1954, JIMT-1, and SKBR3) and triple negative breast cancer cell lines (BT20, HCC1143, HCC1937, MDA-MB-468, MDA-MB-231, and HCC1395). **d.** VWA5A bands in HER2+ breast cancer cell lines (HCC1954, JIMT-1, and SKBR3) and triple negative breast cancer cell lines (BT20, HCC1143, HCC1937, MDA-MB-468, MDA-MB-231, and HCC1395).
